# Clinical and radiological features associated with rupture of pulmonary artery pseudoaneurysm: a retrospective study

**DOI:** 10.1186/s12890-024-03225-0

**Published:** 2024-08-28

**Authors:** Min Liu, Jixiang Liu, Wei Yu, Xiaoyan Gao, Shi Chen, Wei Qin, Ziyang Zhu, Chenghong Li, Fajiu Li, Zhenguo Zhai

**Affiliations:** 1 National Center for Respiratory Medicine, State Key Laboratory of Respiratory Health and Multimorbidity, National Clinical Research Center for Respiratory Diseases, Institute of Respiratory Medicine, Department of Pulmonary and Critical Care Medicine, Center of Respiratory Medicine, Chinese Academy of Medical Sciences, China-Japan Friendship Hospital, No 2, East Yinghua Road, 100029 Beijing, China; 2 Department of Pulmonary and Critical Care Medicine, Affiliated Hospital of Jianghan University, No.168 Hongkong Road, 430000 Wuhan, Hubei China; 3 Institute of Pulmonary Vascular Diseases, Jianghan University, No.168 Hongkong Road, 430000 Wuhan, Hubei China

**Keywords:** Pulmonary artery pseudoaneurysm, Hemoptysis, Rupture, Pulmonary cavitation

## Abstract

**Background:**

Hemoptysis resulting from rupture of the pulmonary artery pseudoaneurysm (PAP) is massive and fatal, while factor contributing to the rupture of pseudoaneurysm remains elusive. This study aimed to elucidate the clinical and radiological features of PAP and identify the risk factors associated with rupture.

**Methods:**

Patients who developed hemoptysis with PAP were collected from January 2019 to December 2022 retrospectively. Clinical data of the demographic characteristics, radiological findings, treatment strategies, and prognosis were collected. A comparative analysis was performed on the characteristics in the ruptured and non-ruptured cases.

**Results:**

A total of 58 PAPs were identified in the 50 patients. The most common causes were infection (86%) and cancer (8%). The PAPs were located predominantly in the upper lobes of both lungs, and 57 (99.3%) were distributed in the segmental or subsegmental pulmonary arteries. The median diameter was 6.1(4.3–8.7) mm. A total of 29 PAPs were identified adjacent to pulmonary cavitations, with the median diameter of the cavity being 18.9 (12.4–34.8) mm. Rupture of pseudoaneurysm occurred in 21 cases (42%). Compared to unruptured group, the ruptured group had a significantly higher proportion of massive hemoptysis (57.1% vs. 6.9%, *p* < 0.001), larger pseudoaneurysm diameter (8.1 ± 3.2 mm vs. 6.0 ± 2.3 mm, *p* = 0.012), higher incidence of pulmonary cavitation (76.2% vs. 44.8%, *p* = 0.027), and larger cavitation diameters (32.9 ± 18.8 mm vs. 15.7 ± 8.4 mm, *p* = 0.005). The mean pulmonary artery pressure (mPAP) in the ruptured group was also significantly higher than that in the unruptured group [23.9 ± 7.4 mmHg vs. 19.2 ± 5.0 mmHg, *p* = 0.011]. Endovascular treatment was successfully performed in all 21 patients with ruptured PAP, of which the clinical success rate was 96.0%. Five patients experienced recurrent hemoptysis within one year.

**Conclusions:**

Massive hemoptysis, pseudoaneurysm diameter, pulmonary cavitation, and elevated mPAP were the risk factors for rupture of pseudoaneurysm. Our findings facilitate early identification and timely intervention of PAP at high risk of rupture.

## Background

Hemoptysis is a serious clinical complication, which can be fatal. The source of hemorrhage is mainly the bronchial artery. Hemoptysis due to pulmonary arterial origin is quite rare and it is estimated to occur in less than 10% of cases [1]. Pulmonary artery pseudoaneurysm (PAP) is one of causes associated with pulmonary artery. Histologically, a pseudoaneurysm comprises either the media, adventitia, or the soft tissue surrounding the vessel, unlike a true aneurysm which involves all three layers of the artery. As a consequence, the pseudoaneurysm has a higher risk of rupture [2]. Hemoptysis has been described as a possible warning sign for rupture of PAP. In patients with massive hemoptysis undergoing bronchial artery embolization, 5-11% patients result from PAP rupture [3]. Once PAP ruptures, the mortality can be as high as 50% [4]. Therefore, risk assessment of the rupture of PAP is critical for early identification.

As low incidence and asymptomatic manifestation, patients with PAP are frequently underdiagnosis or misdiagnosed. PAP may be congenital or acquired. It has been found that PAP was associated with infection, primary or metastatic lung neoplasm, traumatic injury, pulmonary arterial hypertension, or vasculitis [5, 6]. Previous case series have reported that pseudoaneurysms secondary to aspergillus and tumors both tend to be adjacent to cavitary lesion [4, 7]. Rasmussen’s aneurysms were often accompanied by tuberculous cavities, which appeared to be more prone to rupture and cause fatal hemoptysis [4]. However, it remains uncertain whether the cavitary lesions and the rupture are clinically related.

The diameter of abdominal aortic aneurysm (AAA) has been well-established as a crucial determinant and independent predictor of AAA rupture [8]. A reliable assessment of hemodynamics is also crucial for predicting the risk of AAA rupture [9]. Nevertheless, the relationship between pseudoaneurysm size and hemodynamics and PAP rupture has rarely been discussed. Currently, only case reports and series have described the etiology and radiological features. Little is known about the relevant factors for rupture of pseudoaneurysm in the lung. Therefore, this study aimed to illustrate the clinical characteristics of the rupture of pseudoaneurysm in a single center and further identify patients at high risk of rupture for early intervention.

## Methods

### Study population

Clinical data were collected from individuals who underwent computed tomography angiography (CTA) or transcatheter pulmonary vascular intervention for hemoptysis between January 2019 and December 2022. The medical data collected included age, gender, clinical symptoms, medical history, volume of hemoptysis, underlying etiology, imaging characteristics, and intervention and treatment outcomes. Hemoptysis was defined as mild (≤ 39 ml/day), moderate (40–199 ml/day), or massive (≥ 200 ml/day) [10]. The study received approval from institutional review boards for the retrospective review of electronic records and imaging (WHSHIRB-K-2022004). Written informed consent was obtained from each participant.

### Imaging findings

#### Computed tomography angiography

A 64-detector CT scanner was used to conduct CTA, from the thoracic inlet to a position 5–10 cm above the upper abdomen, employing a slice thickness of 1.00 mm. PAP was identified as focal dilations of the pulmonary artery. Information regarding the location, number, size, and level of PAP was collected systematically. Other CT findings, such as cavitations adjacent to PAP were also documented. The size of the PAP was determined using the longest diameter observed on the axial CT scan [11]. The size of the cavitation was measured based on its maximum diameter [12].

#### Digital subtraction angiography

All of the patients underwent bronchial and non-bronchial systemic collateral arterial angiography to identify the target vessels of hemoptysis. Right heart catheterization was conducted during digital subtraction angiography (DSA). This procedure involved measuring pressures at various sites and calculating the cardiac output (CO) using thermodilution. Pulmonary angiography was performed initially at the bifurcation of the right or left pulmonary artery to demonstrate the presence of any PAP and to delineate the anatomy of the pulmonary artery. Selective segmental or subsegmental angiography was performed to determine the location and feeding vessel.

Based on imaging from CTA and DSA, the PAPs were classified into four types. Type A can be visualized by non-selective pulmonary arteriography, type B by selective segmental or subsegmental pulmonary arteriography, type C by bronchial and non-bronchial systemic arteriography, while type D is only visible on pulmonary CT angiography and not on catheter-directed angiography [13]. If a PAP was not visualized on CTA but appeared on DSA pulmonary angiography, the maximum diameter of the PAP observed by DSA was measured to determine its size. The criteria of identification of rupture: (1) the presence of pseudoaneurysm and extravasation of contrast agent in angiography. (2) no signs of bronchial artery rupture were observed during angiography in patients with paroxysmal hemoptysis. (3) cessation or significant reduction of hemoptysis after embolization.

#### Treatment methods

A 5 F angiography catheter (Cook, USA) was superselected into the feeding vessels of the PAP and a 1.98 F microcatheter (Asahi, Japan) was superselected into the pseudoaneurysm sac to embolize with the coil. In a case in which the microcatheter could not reach the aneurysm sac, a pseudoaneurysm was excluded by coil embolization of the feeding vessel. The neck of the pseudoaneurysm was embolized when the pseudoaneurysm sac was too large to embolize fully. Patients were observed for 72 h postoperatively to assess complications and treatment effects. The evaluation of PAP embolization included technical success and clinical success [14]. Technical success was defined as a pseudoaneurysm no longer visible after embolization, while clinical success was defined as cessation of hemoptysis or a significant reduction after the procedure.

### Statistical analyses

All the statistical analyses were conducted using SPSS (version 24.0, IBM Corp). The data were expressed as either median and the first and third quartiles (Q1-Q3), mean ± standard deviation (SD), or absolute number and percentage of patients. For normally distributed data, the t-test was used to compare differences between the two groups. If the data were non-normally distributed, the Mann-Whitney U test was performed. Chi-square (χ2) tests or Fisher exact tests were used to compare proportions between the two groups. Statistical significance was defined as *p* < 0.05.

## Results

### Study population

**Fig. 1:**
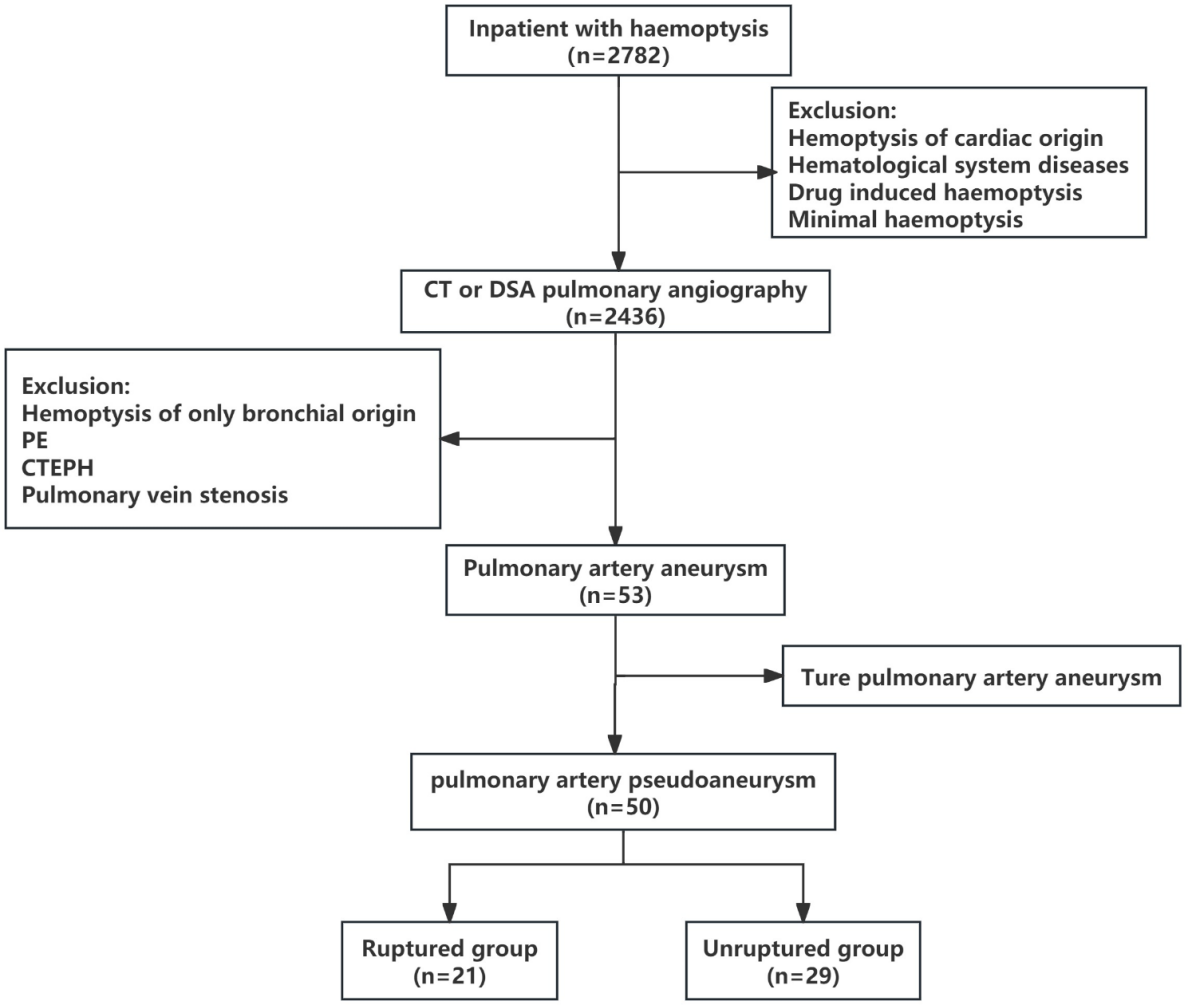
The flowchart of selecting the study population

**Fig. 2:**
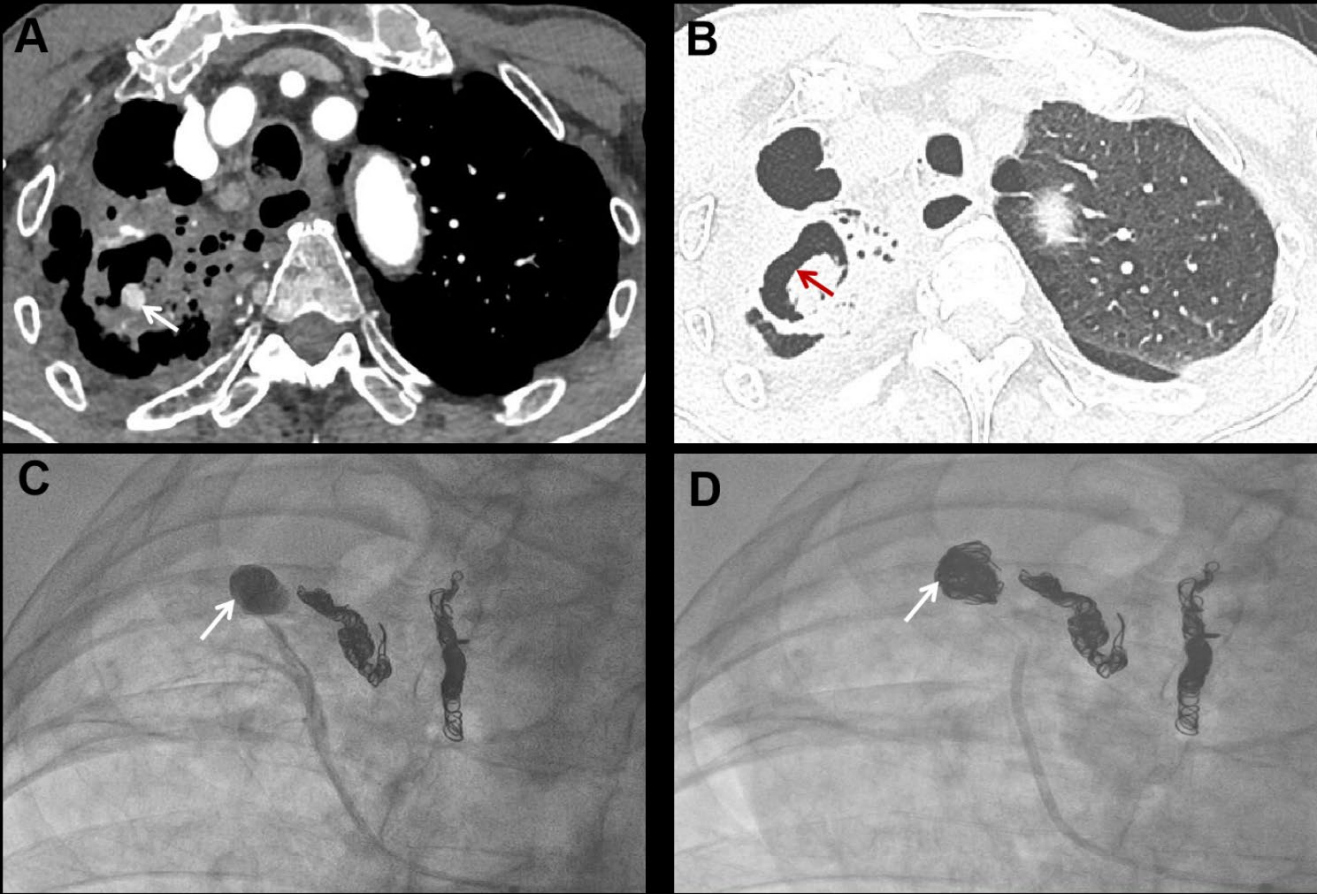
Representative images of Aspergillus infection combined with pulmonary artery pseudoaneurysm. A 48-year-old male was admitted with massive hemoptysis. Sputum culture confirmed aspergillus infection. Bronchial artery embolization was performed six months ago due to hemoptysis. (**A**) The axial enhanced CT scan reveals a PAP (arrow) located in the segmental pulmonary artery of the right upper lobe. The PAP is accompanied by cavitary lesions; (**B**) The axial enhanced CT scan displayed a crescent-shaped cavity (red arrow) in the right upper lobe, indicating a typical imaging manifestation of aspergillus infection; (**C**) Selective subsegmental pulmonary artery angiography confirmed the PAP (arrow); (**D**) The aneurysmal sac (arrow) was embolized with coils

From January 2019 to December 2022, a total of 2782 inpatients presented with hemoptysis. PAP was detected in 50 cases by pulmonary artery CTA or DSA arteriography. The flowchart for selecting the study population is shown in Fig. 1. The baseline characteristics of these patients are summarized in Table 1. The mean age was 62.1 ± 13.4 years, with 36 (72%) of them being male. The proportion of patients with massive hemoptysis was 14 (28%), and three of them received preoperative endotracheal intubation due to asphyxia. The cause of PAP is listed in Table 2. Notably, the most common causes were infection and pulmonary malignancy. Infection was presented in 43 cases (86%), including obsolete and active tuberculosis (40% and 12%, respectively), bronchiectasis (12%), and focal pneumonia (8%). Fungal infection was observed in 5 patients (10%) and 3 of them were identified as Aspergillus infection through sputum culture (Fig. 2). Four cases (8%) were caused by malignant tumors, including primary lung cancer and liver cancer with lung metastasis.

**Table 1:** Baseline characteristics of pulmonary artery pseudoaneurysm in 50 patients with hemoptysis

Parameters	Case Number
**Age**,** (years)**	62.1 ± 13.4
**Male**,** n(%)**	36 (72)
**Signs and symptoms**	
Heart rate (bpm)	85.6 ± 12.8
Systolic blood pressure (mmHg)	122.9 ± 15.3
Diastolic blood pressure, (mmHg)	75.9 ± 10.7
Respiratory rate, (times/min)	20 ± 2.0
Fever	3 (6)
Cough	9 (18)
Dyspnea	4 (8)
Chest tightness	7 (14)
Disorders of consciousness	1 (2)
**Hemoptysis**,** n(%)**	
Mild	16 (32)
Moderate	20 (40)
Massive	14 (28)
**Comorbidities**,** n(%)**	
Chronic obstructive pulmonary disease	7 (14)
Pneumoconiosis	3 (6)
Prior thoracic surgery	5 (10)
Hypertension	9 (18)
Coronary heart disease	2 (4)
Diabetes mellitus	9 (18)

**Table 2:** Causes of pulmonary artery pseudoaneurysms in patients with hemoptysis

Underlying disease	Case Number
**Infection**,** n(%)**	43 (86)
Tuberculosis	26 (52)
Bronchiectasis	6 (12)
Fungal pneumonia	5 (10)
Focal pneumonia	4 (8)
Lung abscess	2 (4)
**Malignant tumor**,** n(%)**	4 (8)
Lung adenocarcinoma	1 (2)
Lung squamous cell carcinoma	2 (4)
Pulmonary metastasis	1 (2)
**Percutaneous lung biopsy**	1 (2)
**Congenital heart disease**,** n(%)**	1 (2)
**Pulmonary fibrosis**,** n(%)**	1 (2)

### Imaging features

**Fig. 3:**
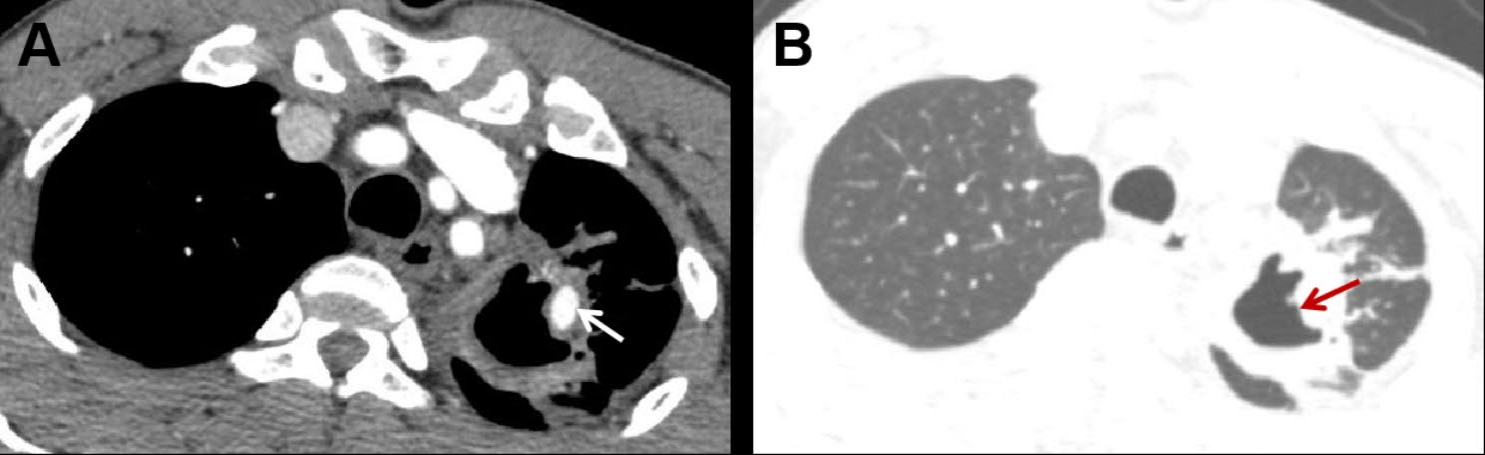
Representative images of tuberculous cavity combined with pulmonary artery pseudoaneurysm. A 54-year-old male was admitted to the hospital due to massive hemoptysis. He was diagnosed with sputum smear-positive pulmonary tuberculosis. (**A**) Axial contrast-enhanced CT scan shows a pseudoaneurysm (arrow) in the segmental pulmonary artery of the left upper lobe; (**B**) Axial contrast-enhanced CT scan shows a large cavitary lesion (red arrow) adjacent to the PAP

**Fig. 4:**
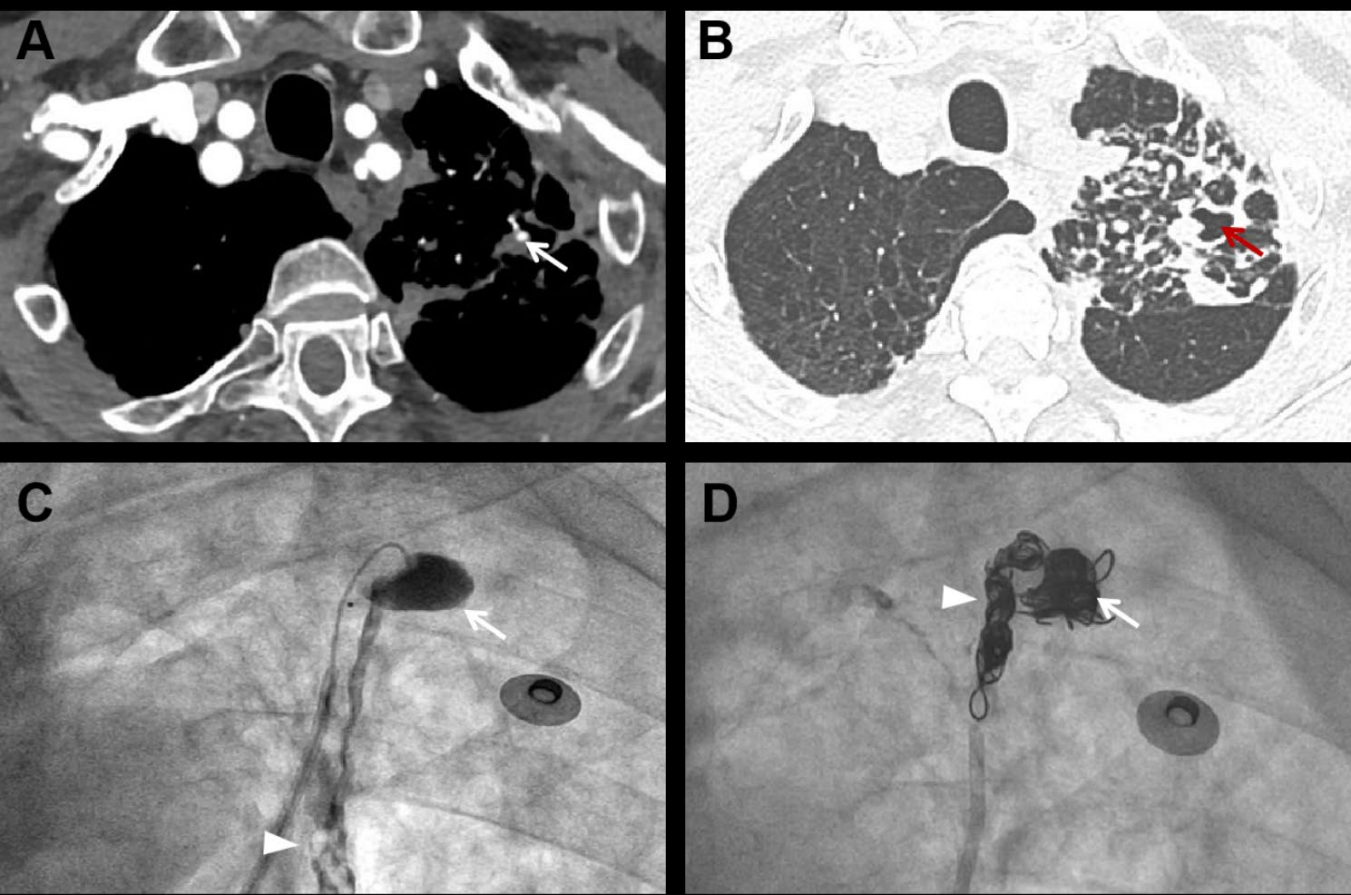
Representative images of ruptured pulmonary artery pseudoaneurysm. A 76-year-old male was hospitalized due to sudden massive hemoptysis. The patient had a history of tuberculosis and a positive sputum smear indicated the recurrence of tuberculosis. (**A**) Axial contrast-enhanced CT scan shows a pseudoaneurysm (arrow) distributed in the segmental pulmonary artery of the left upper lobe; (**B**) Axial contrast-enhanced CT scan shows that the PAP is adjacent to a cavitary lesion (red arrow); (**C**) Selective subsegmental pulmonary arteriography shows contrast agent extravasation from the PAP to the cavity (arrow) and trachea (arrowhead), confirming its rupture; (**D**) Final angiography shows occlusion of pseudoaneurysm (arrow) and the proximal of the feeding vessel (arrowhead) after coil embolization

A total of 58 PAPs were identified in all the patients with hemoptosis. The imaging features are detailed in Table 3. The PAPs were located primarily in the upper lobes of the lungs, with 24 (41.4%) in the left upper lobe, and 18 (31.0%) in the right upper lobe. They had a strong predilection for the peripheral pulmonary arteries and 57 (99.3%) were located in the segmental or subsegmental pulmonary arteries. The median diameter of PAP was 6.1(4.3–8.7) mm, and the maximum diameter was 15.2 mm. A total of 29 PAPs were identified adjacent to pulmonary cavitations (Fig. 3). The median diameter of the cavity was 18.9 (12.4–34.8) mm, with the maximum diameter being 77.4 mm. Four cases (8%) coexisted with bronchial artery aneurysms. pseudoaneurysm in 21 cases (42%) were considered as ruptured, of which most presented massive hemoptysis (Fig. 4).

**Table 3:** Imaging features and treatment of 58 pulmonary artery pseudoaneurysms in patients with hemoptysis

Imaging features and treatment	Value
**Side**, ***n***(%)	
Left	29 (50)
Right	29 (50)
**Lobar distribution**,** n(%)**	
Left upper lobe	24 (41.4)
Left lower lobe	5 (8.6)
Right upper lobe	18 (31.0)
Right middle lobe	3 (5.2)
Right lower lobe	8 (13.8)
**Level of distribution**,** n(%)**	
Lobar	1 (1.7)
Segmental	11 (20.0)
Subsegmental	46 (79.3)
**Classification**,** n**	
A/B/C/D	0/31/25/2
**Maximum diameter of PAP (mm)**	6.1 (4.3–8.7)
**Ruptured pseudoaneurysms**,** n (%)**	21 (42)
**Cavitary lesion**,** n(%)**	29 (50)
**Maximum diameter of cavitary lesion (mm)**	18.9 (12.4–34.8)
**The site of embolization**	
Aneurysmal sac	13 (61.9)
Aneurysmal neck	1 (4.8)
Feeding vessel	10 (47.6)
**Embolization materials**,** n (%)**	
Coil	20 (95.2)
Covered stent	1 (4.8)

### Clinical characteristics of ruptured pulmonary artery pseudoaneurysm

To identify the risk factors for rupture of PAP, we compared the clinical features between ruptured and unruptured patients. The clinical characteristics and hemodynamic features of the two groups are summarized in Table 4. No significant differences were observed between the two groups across gender, age, etiology, and distribution. Compared to the non-rupture group, the rupture group had a significantly higher proportion of massive hemoptysis (57.1% vs. 6.9%, *p* < 0.001), larger pseudoaneurysm diameter (8.1 ± 3.2 mm vs. 6.0 ± 2.3 mm, *p* = 0.012), higher incidence of pulmonary cavitation (76.2% vs. 44.8%, *p* = 0.027), and larger cavitation diameters (32.9 ± 18.8 mm vs. 15.7 ± 8.4 mm, *p* = 0.005). The mean pulmonary artery pressure (mPAP) in the ruptured group was also significantly higher than that in the unruptured group (23.9 ± 7.4 mmHg vs. 19.2 ± 5.0 mmHg, *p* = 0.011). Meanwhile, there was no statistical significance in the other hemodynamic parameters.

**Table 4:** Comparison of clinical characteristics of patients with ruptured and unruptured pulmonary artery pseudoaneurysms

Characteristics	Ruptured group (*n* = 21)	unruptured group (*n* = 29)	*P* value
**Age**,** (years)**	61.3 ± 10.2	62.6 ± 15.5	0.748
**Male**,** n(%)**	18 (85.7)	18 (62.1)	0.066
**Massive hemoptysis**,** n(%)**	12 (57.1)	2 (6.9)	<0.001
**Underlying disease**,** n(%)**			
Tuberculosis	12 (57.1)	14 (48.3)	0.536
Bronchiectasis	2 (9.5)	4 (13.8)	0.986
Fungal pneumonia	3 (14.3)	2 (6.9)	0.702
Malignant tumor	1 (4.8)	3 (10.3)	0.849
Location of PAP*,** n(%)**			
Total	26	32	
Left	11 (42.3)	18 (56.3)	0.291
Right	15 (57.7)	14 (43.7)	
Upper lobes	23 (88.5)	22 (68,7)	0.073
Lower lobes	3 (11.5)	10 (31.3)	
Segmental	7 (26.9)	4 (12.5)	0.184
Subsegmental	18 (69.2)	28 (87.5)	
**Maximum diameter of PAP (mm)**	8.1 ± 3.2	6.0 ± 2.3	0.012
**Cavitary lesion**,** n(%)**	16 (76.2)	13 (44.8)	0.027
**Maximum diameter of cavitary lesion (mm)**	32.9 ± 18.8	15.7 ± 8.4	0.005
**Hemodynamics**			
CO (L/min)	5.5 ± 0.9	5.3 ± 1.4	0.570
CI (L/min/m2)	3.5 (3.3-4.0)	3.2 (2.8-4.0)	0.232
mPAP (mmHg)	23.9 ± 7.4	19.2 ± 5.0	0.011
PAWP (mmHg)	11.8 ± 4.1	10.0 ± 3.8	0.137
PVR (Wood units)	2.1 (1.3-3.0)	1.8 (1.4–2.3)	0.647

Abbreviations: CO, cardiac output; CI, cardiac index; mPAP, mean pulmonary artery pressure; PAWP, pulmonary artery wedge pressure; PVR, pulmonary vascular resistance

* The analyses of location included 26 PAPs in ruptured group and 32 PAPs in unruptured group

### Treatment and outcome

Endovascular treatment was conducted in all patients with PAP rupture. As shown in Table 2. Coil embolization was performed in 20 cases (95.2%), including pseudoaneurysm sac embolization in 13 cases (61.9%), pseudoaneurysm neck embolization in 1 case (4.8%), feeding vessel embolization in 10 cases (47.6%). One PAP was excluded with covered stents. No patient underwent surgical resection. The outcome and complication of patients with PAP rupture are listed in Table 5. During the operation, one patient experienced stress-induced hypertension, and another one sustained a pulmonary artery injury. One patient suffered massive hemoptysis due to rupture of PAP during pulmonary angiography, and the hemoptysis stopped after immediate embolization. After the operation, fever, chest tightness, and chest pain were complained by patients with interventional treatment. All of the complications resolved spontaneously or following symptomatic treatment. None of the patients had pulmonary infarction. The technical and clinical success rate was 100% and 96.0%, respectively. One patient died of massive hemoptysis during hospitalization.

Of the patients received intravascular intervention for patients with PAP rupture, 17 patients completed one-year follow-up. One patient died of heart failure. Meanwhile, five patients had recurrences of hemoptysis, of which four occurred within 6 months.

**Table 5:** Outcome and complications of patients with ruptured pulmonary artery pseudoaneurysm

Parameters	Case Number
**Outcome**	
Technical success	21 (100)
Clinical Success	19 (90.5)
**Intraoperative complication**	
Stress-induced hypertension	1 (4.8)
Aneurysm rupture	1 (4.8)
Pulmonary artery injury	1 (4.8)
**Postoperative complication**	
Fever	2 (9.5)
Chest tightness	1 (4.8)
Chest pain	1 (4.8)

## Discussion

This study described the clinical and imaging features of patients with hemoptysis and PAP, and further investigated the risk factors for rupture of PAP. The findings of this study indicated that massive hemoptysis, pseudoaneurysm diameter, cavitary lesions, and elevated mPAP were associated with an increased risk of PAP rupture. To our knowledge, this is the first study identifying risk factors for rupture of pseudoaneurysm. This study could assist clinicians in identifying high-risk patients of PAP rupture, offering insights into treatment strategies that could prevent delays in critical interventions.

The mechanism of PAP formation is vessel wall destruction and further replaced by granulomatous, neoplastic, or fibrotic tissue, resulting in the weakening of the arterial wall. Under sustained arterial pressure, blood dissects the injured artery and forms a perfused sac. Infection was the most common cause of acquired PAP in our patient population, which is consistent with previous studies [5, 6]. Previous studies have reported that PAPs were often accompanied by cavitary lesions [3, 4, 7]. It revealed that PAP may have a predilection for pulmonary cavitation. In our study, PAPs were adjacent to pulmonary cavities in 29 patients (58%). Compared with the unruptured patients, the ruptured ones had a significantly higher proportion of pulmonary cavitation and the size of cavitary lesions was larger.

The underlying pathogenesis may be direct invasion of vessels by pathogenic bacteria and persistent damage by pulmonary lesions associated with tissue necrosis or inflammation. Cystic medial necrosis was observed in many perioperative samples of the vascular wall [15]. Inflammation has been considered as a central driving cause in the development of arterial aneurysm. Studies have demonstrated that Chlamydia pneumonia is detected in AAA with infiltration of inflammatory cells including macrophages, lymphocytes, and plasma cells in the aortic walls [16–18]. In terms of lung infection, patients with tuberculosis, necrotizing pneumonia, and suppurative bacterial and fungal infections were more likely to have PAP [19]. The common causative organisms of mycotic PAP were Staphylococcus and Streptococcus, especially in patients with infective endocarditis [20, 21]. Of note, the vascular wall in the pulmonary circulation was destructed with the formation of PAP through hematogenous dissemination or direct spread from the pulmonary infectious focus. These patients often had a history of intravenous drug abuse and presented with fever, bacteremia, and massive hemoptysis, requiring intravascular interventional therapy [22]. Therefore, it is reasonable to infer that pulmonary cavities may be associated with the formation and rupture of PAP.

The degeneration of the pathological structure is not sufficient to produce an abnormal balloon shape. The formation and rupture of this abnormal structure are also influenced by intravascular hemodynamic effects in addition to the weakened arterial wall. In an observational study about a cohort of patients with pulmonary hypertension, the incidence of pulmonary artery aneurysm was about 38%, especially in patients with long disease duration [23]. In patients with hemoptysis due to PAP rupture, especially those with systemic-pulmonary shunts, pulmonary artery pressure is maintained between 35 and 69 mmHg [4]. Our data indicated that PAP in the ruptured group exhibited larger diameter and higher mPAP in comparison to the unruptured group. Similar to AAA, vessel wall tension escalates with both diameter and transmural pressure [24]. Arterial wall tension is directly proportional to both pressure and the radius of the diseased vessel while inversely correlated with arterial wall thickness according to the Laplace’s law. Rupture occurs when stress induced by blood flow exceeds the limit of wall tissue strength. Thus, risk of rupture in the PAP is closely associated with both size and pressure.

There are no guidelines for the indication of treatment and the best therapeutic approach. The main treatment for PAP were surgery and intravascular intervention. Surgical treatments such as lobectomy or aneurysmectomy were associated with high mortality [25]. Endovascular coil embolization and stent placement are currently preferred due to less invasive and fewer complications [26, 27]. The selection of embolization material depends on the location, size, and classification of PAP. Coil embolization may be an effective choice for preserving distal pulmonary artery perfusion and avoiding lung perfusion injury. The stent is generally recommended for fusiform or lobar arterial pseudoaneurysm in the pulmonary. Of the current study, most of the patients received coil embolization and only one PAP located in the lobar branch was excluded with a covered stent. Due to vulnerability to rupture of PAP, the operator should be alert to sudden rupture during the operation [28]. In our series, one rupture occurred during pulmonary angiography. The possible reason might be a transient increase in pulmonary perfusion and local pressure with contrast agent injection, or direct damage to microcatheters and microwires during the procedure. In addition, some infectious PAP is curable. The conservative treatment with intravenous antibiotics alone has been documented to be successful in patients with small and stable aneurysms [29].

We acknowledge several limitations of our study. First, although our study cohort was relatively large compared to the previous studies, we still did not include all causes of PAP, such as endocarditis due to the limited cases. Furthermore, the natural history of PAP has not yet been extensively studied and remains largely unknown. Experts evaluated the cause of PAP based on the history and imaging of patients. Thus, its accuracy and conclusiveness may be limited. Additionally, longer-term follow-up studies are crucial for evaluating the extended prognosis of patients with PAP and hemoptysis.

## Conclusions

In conclusion, we found that PAP patients with rupture had a significantly higher proportion of massive hemoptysis and pulmonary cavitation, larger pseudoaneurysm and cavitation diameter, as well as higher mPAP. Collectively, we have characterized the clinical features of PAPs and explored risk factors of rupture, providing novel insights into the early identification of PAPs with high risk of rupture.

## Data Availability

The data used and analyzed in the study are available from the corresponding author on reasonable request.

## Abbreviations

PAP: Pulmonary artery pseudoaneurysm
AAA: Abdominal aortic aneurysm
CTA: Computed tomography angiography
DSA: Digital subtraction angiography
CO: Cardiac output
CI: Cardiac index
mPAP: Mean pulmonary artery pressure
PAWP: Pulmonary artery wedge pressure
PVR: Pulmonary vascular resistance
